# Protocol for a systematic review and meta-analysis of the prevalence of mental illness among nursing home residents

**DOI:** 10.1186/s13643-024-02516-1

**Published:** 2024-04-16

**Authors:** Jared Holt, Sunil Bhar, Penelope Schofield, Deborah Koder, Patrick Owen, Dallas Seitz, Jahar Bhowmik

**Affiliations:** 1 Department of Psychological Sciences, Swinburne University of Technology, John Street, Hawthorn, VIC 3122 Australia; 2 Iverson Health Innovation Research Institute, Swinburne University of Technology, John Street, Hawthorn, VIC 3122 Australia; 3 Health Services Research and Implementation Sciences, Peter MacCallum Cancer Centre, 305 Grattan Street, Melbourne, VIC 3052 Australia; 4 Department of Oncology, Sir Peter MacCallum, The University of Melbourne, Grattan Street, Parkville, VIC 3010 Australia; 5 Eastern Health Emergency Medicine Program, Melbourne, VIC Australia; 6 Eastern Health Clinical School, Monash University, Melbourne, VIC Australia; 7 Department of Psychiatry, Cumming School of Medicine, University of Calgary, 3330 Hospital Drive NW, Calgary, AB T2N 4N1 Canada; 8 Department of Health Science and Biostatistics, Swinburne University of Technology, John Street, Hawthorn, VIC 3122 Australia

**Keywords:** Nursing homes, Older adults, Mental illness, Prevalence, Systematic review, Meta-analysis, Protocol

## Abstract

**Background:**

There is a high prevalence of mental illness in nursing home residents compared to older adults living in the community. This was highlighted in the most recent comprehensive systematic review on the topic, published in 2010. In the context of a rapidly aging population and increased numbers of older adults requiring residential care, this study aims to provide a contemporary account of the prevalence of mental illness among nursing home residents.

**Methods:**

This protocol was prepared in line with the PRISMA-P 2015 Statement. Systematic searches will be undertaken across six electronic databases: PubMed, Embase, Web of Science, PsycNET, CINAHL, and Abstracts in Social Gerontology. Peer-reviewed studies published from 2009 onwards which report the prevalence of mental illness within nursing home populations will be included. Database searches will be supplemented by forward and backward citation searching. Titles and abstracts of records will be screened using a semi-automated process. The full text of selected records will be assessed to confirm inclusion criteria are met. Study selection will be recorded in a PRISMA flowchart. A pilot-tested form will be used to extract data from included studies, alongside the JBI Critical Appraisal Checklist for Studies Reporting Prevalence Data. A study characteristics and results table will be prepared to present key details from each included study, supported by a narrative synthesis. Random-effects restricted maximum likelihood meta-analyses will be performed to compute pooled prevalence estimates for mental illnesses represented in the identified studies. Heterogeneity will be assessed using Cochran’s Q and Higgins’ *I*^*2*^ statistics. A Funnel plot and Egger’s test will be used to assess publication bias. The GRADE approach will be used to assess the quality of the body of evidence identified.

**Discussion:**

The study will provide a comprehensive and contemporary account of the prevalence of mental illness among nursing home residents. Meta-analyses will provide robust prevalence estimates across a range of presentations. Key insights will be highlighted, including potential sources of heterogeneity. Implications for residents, researchers, care providers, and policymakers will be noted.

**Systematic review registration:**

PROSPERO: CRD42023456226.

**Supplementary Information:**

The online version contains supplementary material available at 10.1186/s13643-024-02516-1.

## Background

The world’s population is aging at an increasing rate. The number of individuals aged 60 and over is predicted to double by 2050 and the population aged 80 and over is expected to triple [1]. While longer lifespans afford additional opportunities to individuals and societies, they also introduce challenges in managing the burden of disease associated with aging.

Nursing homes are residential facilities that provide care for older adults and other disabled individuals whose care needs are unable to be met in their own homes. Understanding the needs of nursing home residents is a necessary precondition to ensure systems are appropriately designed and resourced. This becomes even more important considering the number of nursing homes (and residents) will inevitably increase alongside the aging population.

Unfortunately, relatively little is known about the prevalence of mental illness among nursing home residents. The last comprehensive systematic review on the topic was published in 2010 [2]. The authors found dementia, depression, and anxiety disorders to be the most common mental illnesses among older adults in long-term care. However, the authors did not undertake meta-analyses to compute pooled prevalence estimates for the illnesses and reported median figures only. A dearth of prevalence studies addressing other common mental illnesses (e.g., anxiety disorders, schizophrenia, and bipolar disorder) in the nursing home population was also noted by Seitz and colleagues. The authors further commented that many of the studies included in their paper may not accurately reflect present-day prevalence rates due to their age (more than half of the studies were published prior to 2000). This issue has only been exacerbated given the ever-changing landscape of an aging population [1], as well as advancements in how mental illnesses are understood and related refinements to diagnostic criteria and instruments [3]. Furthermore, there has been considerable methodological progress regarding the conduct of systematic reviews since the early 2000s and updated guidance to ensure greater robustness, reliability, and transparency [4–6].

Elias [7] and Fornaro and colleagues [8] both carried out more recent targeted reviews. The former included loneliness, anxiety, and depression, while the latter considered major depressive disorder, bipolar disorder, and schizophrenia. However, neither study provided a rationale for the selection of the chosen disorders, nor the exclusion of others. Fornaro and colleagues [8] further restricted their inclusion criteria to only consider studies investigating nursing home residents without dementia. This decision acts to critically limit the external validity of their findings. A recent meta-analysis of the prevalence of dementia in long-term care institutions found that more than half of all residents live with dementia [9]. Given dementia appears to be the rule rather than the exception in this population, residents with comorbid dementia must be considered if prevalence estimates are to be of use to decision-makers.

The present study builds on and expands the previous reviews to provide a contemporary and comprehensive account of the prevalence of mental illness among nursing home residents. It is not merely intended as an update of Seitz and colleagues’ paper [2]. This study will leverage the considerable methodological progress and guidance on conducting systematic reviews that have been published since Seitz and colleagues released their study in 2010. In doing so, it aims to generate rigorous and reliable estimates of mental illness prevalence in nursing homes.

As compared to recent reviews (e.g., [7, 8]), this study will address a much broader range of mental illnesses. It will also better reflect the realities and complexities of the nursing homes, particularly through ensuring dementia co-morbidities are duly considered. In doing so, we hope to provide nursing home organizations, researchers, and governments with the necessary evidence to inform planning efforts and ensure the mental health needs of this vulnerable population can be met.

## Methods/design

This protocol is for a systematic review and meta-analysis of mental illness among nursing home residents. It was registered on PROSPERO on 01 September 2023 (CRD42023456226). Any future updates to the protocol will be reflected in the PROSPERO registration. The protocol has been informed by the Preferred Reporting Items for Systematic Reviews and Meta-Analyses Protocols (PRISMA-P) 2015 Statement [10] and used the PRISMA-P 2015 checklist (see Additional file 1). The systematic review and meta-analyses will be undertaken in alignment with the relevant chapter of the JBI Manual for Evidence Synthesis [4].

### Eligibility criteria

The systematic review and meta-analysis will consider studies measuring the prevalence of mental illness among nursing home residents, published from 01 January 2009. The publication date of these studies aligns with the end-point of the last comprehensive systematic review of the topic [2] and ensures a focus on the modern-day nursing home experience. Non-English publications will be considered where abstracts are available in English and the information required for data extraction is provided.

The ‘CoCoPop’ mnemonic (Condition, Context, and Population; [4]) has been used to guide inclusion requirements. That is, for studies to be included they must consider the relevant condition (mental illness), be presented in the appropriate context (prevalence rates), and apply to the target population (nursing home residents).

#### Condition (mental illness)

Studies investigating at least one mental disorder as defined by the Diagnostic and Statistical Manual of Mental Disorders [11–14] or the International Classification of Diseases and Related Health Problems [15, 16] will be considered for this review. Studies investigating clinically significant mental disturbances or symptoms will also be included where measures have been validated for target conditions and cut-off scores have been established to indicate clinical significance (e.g., scores of eight or more on the Cornell Scale for Depression in Dementia; [17]). Noting the prevalence of dementia in nursing homes has been addressed in a recent systematic review [9], dementia diagnoses will not be separately considered in this study. However, comorbid dementia will be considered where study authors have reported this information in the context of additional mental disorder diagnoses or clinically significant symptomology.

#### Context (prevalence)

The systematic review and meta-analyses will include peer-reviewed observational epidemiological studies that focus on identifying the prevalence of mental illness (mental disorders or clinically relevant symptoms), including cross-sectional studies, retrospective cohort studies, and prospective longitudinal cohort studies. For longitudinal studies, point/period prevalence estimates will be taken from the first reported time-period. Validation studies will also be considered where tools with established validity have been used as comparators and relevant statistics have been reported. Other study designs not mentioned above, including intervention studies, systematic reviews, case studies, case–control studies, opinion pieces, editorials, etc., will not be considered.

#### Population (nursing homes)

The systematic review will include studies relating to residents of nursing homes, which are variously referred to in the literature as homes for the aged, long-term care, aged care homes, residential aged care facilities, specialized nursing facilities, institutionalized elderly, or institutionalized older adults. Despite nursing homes being largely associated with older adult populations, they are increasingly being used to care for younger individuals [18]. Accordingly, and to ensure a comprehensive and contemporaneously relevant review, no age-based restrictions will be applied. Studies investigating older adults living in the community, retirement homes, or hospital in-patient settings will not be considered. Studies involving mixed populations (e.g., older adults from both community and nursing home settings) will also be excluded unless the groups are separately reported. Additionally, studies focusing on sub-populations or specifically targeted samples will be excluded. For example, this may be samples of nursing home residents who have been ‘pre-screened’ for mental illness, samples of residents suffering from comorbid primary disorders, or studies conducted in psychiatric nursing homes.

### Search strategy

Searches will be conducted across six databases, including PubMed, Embase (Ovid), Web of Science (Clarivate), PsycINFO (APA PsycNet), CINAHL (EBSCOhost), and Abstracts in Social Gerontology (EBSCOhost). The databases were selected based on guidance from Bramer et al. [19] regarding optimal database combinations for literature searches in systematic reviews. Searches will be undertaken in August 2023 and will be re-run in April 2024, ahead of final analyses.

Search queries were developed to operationalize the CoCoPop elements outlined above. As the present study is interested in all mental disorders and clinically relevant symptomology, a broad range of terms were derived from diagnoses contained in the Diagnostic and Statistical Manual of Mental Disorders, Fifth Edition, Text Revision (DSM-5-TR; [14]). Acknowledging the variation in terminology used to describe nursing homes in the literature, search terms also include ‘homes for the aged’, ‘long-term care’, ‘residential aged care’, ‘skilled nursing facilities’, and ‘institutionalized older people’, as well as grammatical variants. ‘Incidence’ and ‘epidemiology’ are similarly included as likely alternatives to prevalence. Search queries require the presence of all three CoCoPop elements, effected through the application of the Boolean ‘AND’ operator. Individual search queries use a combination of MeSH/index terms and text-string searches, depending on the available functionality of each database. Search queries for each of the six databases are provided in Additional file 2. The search strategy was developed in collaboration with and was peer-reviewed by, an information specialist located at the Swinburne University of Technology, Melbourne, Australia.

Forward and backward citation searches will be carried out on previously published reviews to detect additional potentially relevant studies.

### Data management

An online tool will be used to manage the selection and review process. Rayyan is a web-based application for managing systematic reviews which can be accessed for free [20]. Rayyan has been found to have high useability ratings and superior performance in deduplication and software-assisted screening processes [21]. Results from database searches will be imported into Rayyan using compatible file types (.ris or.nbib). Deduplication of records will be undertaken in Rayyan, given its demonstrated accuracy in this process [22]. Extracted data from included studies will be collated in Microsoft Excel and meta-analyses performed in IBM SPSS Statistics (Version 28). Grading of Recommendations Assessment, Development and Evaluation (GRADE; [23]) tables will be prepared using the web-based application, GRADEpro (https://www.gradepro.org/).

### Study selection

Following deduplication, a semi-automated process for screening titles and abstracts will be conducted. Semi-automated processes have been found to have reached maturity with respect to abstract screening in systematic reviews [24]. They offer the potential for significant savings in time and effort while retaining acceptable specificity and sensitivity [24]. However, there remains a lack of consensus regarding recommended ‘stopping rules’. That is, the point at which duplicate human screening can be discontinued and the remaining records subjected to a more streamlined review process [25–27]. Acknowledging this limitation, recent guidance suggests the application of multiple conservative approaches to stopping rules to ensure reliable performance [28].

Rayyan has built-in capability to facilitate semi-automated title and abstract screening (20). Once enough manual decisions have been recorded (at least 50), Rayyan uses machine learning and artificial intelligence to predict the likelihood that each remaining article should be included in the systematic review. The computed likelihoods are presented through a five-star rating system. Each record is assigned either 0.5, 1.5, 2.5, 3.5, or 4.5 stars, with 4.5 stars representing the greatest likelihood of inclusion and 0.5 stars the least. Each subsequent decision made by reviewers provides further guidance for Rayyan’s algorithm and star ratings can be recomputed periodically to generate updated predictions. Records can be sorted from highest to lowest ratings, allowing reviewers to manually screen those records predicted as the most likely to fit inclusion criteria. The process continues until a pre-defined stopping rule is met. This type of approach has been adopted in several recent systematic reviews considering the experiences of older adults [29–31], as well as broader populations [32, 33]. Compared to these recent publications, this study will apply more conservative stopping rules to minimize the risk of missing potentially relevant studies.

Rayyan allows reviewers to assign the labels of ‘include’, ‘exclude’, or ‘maybe’ to each record. Reviewers will be blinded to each other’s decisions until all required records have been reviewed in duplicate. Any disagreements and ‘maybes’ will be resolved via adjudication from a third independent team member. In the present study, the titles and abstracts of records will be independently reviewed by at least two reviewers until both of the following stopping rules have been met: (i) a minimum of 50% of records have been reviewed, and (ii) 100 consecutive articles have been excluded (see [28]). All remaining records will be screened by one reviewer only.

In practice, 10% of retrieved records will be randomly selected and screened by two independent reviewers, at which point Rayyan star ratings will be computed. Records will then be sorted by their star ratings, from highest to lowest. The screening will continue sequentially, starting with the highest-rated record. Ratings will be recomputed when 20% and 40% of records have been screened by two independent reviewers. Records will be re-ordered by rating, from highest to lowest, following each computation. Screening will continue until at least 50% of records have been screened and 100 consecutive records have been excluded. All remaining records will be screened by one reviewer only.

In the next phase, the full text of all records selected to progress (i.e., those ‘included’ during title/abstract review) will be independently reviewed by at least two reviewers, who are blinded to each other’s decisions. This phase will also be facilitated by Rayyan, which again provides reviewers the option to assign the labels of ‘include’, ‘exclude’, or ‘maybe’ to each record. Any disagreements and ‘maybes’ will be resolved via adjudication from a third independent team member.

### Data extraction

Data will be extracted from studies that are confirmed to meet the inclusion criteria via full-text review. A pre-piloted form has been developed, based largely on the Data Extraction Form for Prevalence Studies from Munn et al. ([4]; see Additional file 3). Extracted data items will include general information about the study (e.g., author details, study title, publication year, location); information about the study methods (e.g., study design, sample size, population, inclusion/exclusion criteria, diagnostic criteria/instrument used); and the results (prevalence rates, confidence intervals and/or standard errors, etc.). Data extraction will be undertaken in duplicate by two independent extractors. Any disagreements between the two reviewers will be resolved via adjudication from a third independent team member.

Attempts will be made to obtain any missing data by directly contacting the relevant studies’ investigators. Three attempts will be made to contact each author over a 2-week period. If no response is received, the related study will be excluded from further analysis.

### Risk of bias in individual studies

The JBI Critical Appraisal Checklist for Studies Reporting Prevalence Data [4] will be used to undertake quality and bias assessments of included studies. It is the most recent and methodologically rigorous assessment tool for prevalence studies [34, 35]. Each study will be independently evaluated by at least two reviewers. Any disagreements between the two reviewers will be resolved via adjudication from a third independent team member.

### Data synthesis

A study characteristics and results table will be prepared to present key details from each study that satisfies the inclusion criteria. A narrative synthesis will be undertaken to summarise the relevant studies. This will be supported by meta-analyses to compute pooled prevalence estimates for each mental disorder represented in the identified studies. Specifically, random-effects restricted maximum likelihood meta-analyses will be undertaken given the expected heterogeneity (e.g., study samples will be drawn from different national populations; see [36, 37]). Where meta-analyses include less than five studies, the Hartung-Knapp-Sidik-Jonkman method will be employed per recommendations [38].

Consistent with previous reviews, findings will be presented by disorder subgroups contained in the DSM-5-TR (e.g., depressive disorders, anxiety disorders, personality disorders, feeding and eating disorders; [14]). Findings will also report comparisons between groups with and without dementia, where such comorbidity information is available. Subgroup analyses will apply a series of random-effects models as described above and Cochran’s *Q* and Higgins’ *I*^*2*^ will be used to test for heterogeneity among different subgroups [39, 40]. The subgroup analyses will consider possible differences based on age (less than 65; 65 to 74; 75 to 84; over 85), gender (male; female; non-binary), location (by continent), study design (cross-sectional; prospective longitudinal; retrospective; validation study), and diagnostic tools applied (e.g., for depression this will consider the Geriatric Depression Scale [41]; Cornell Scale for Depression in Dementia [42]; Patient Health Questionnaire [43, 44]; Other). Reflecting concerns regarding underpowered subgroup analyses in meta-analyses [45], subgroup analyses will be undertaken only if 10 or more studies are available and each subgroup contains a minimum of three studies [46].

### Meta-bias(es)

Cochran’s *Q* and Higgin’s *I*^*2*^ statistics will be used to assess statistical heterogeneity [39, 40]. When three or more studies are available, the heterogeneity of observed effects will be evaluated by prediction interval [47]. When 10 or more studies are available, publication bias will be assessed via visual inspection of funnel plots and Egger’s *P* [48].

### Confidence in cumulative evidence

The overall quality of the body of evidence identified through this systematic review will be summarized and assessed using the Grade approach [23]. Although specific guidance is lacking on the application of GRADE to systematic reviews of prevalence, it remains the recommended approach, noting some translatable guidance is available [5, 49].

## Discussion

The present proposed review provides an opportunity to update the literature on the prevalence of mental illness in one of the most vulnerable populations: nursing home residents. This is long overdue with the most recent comprehensive review published in 2010 [2]. It found the population experiences mental illness at significantly higher rates compared to older adults in community settings. The present review will consider the substantial literature published in the intervening period to provide an up-to-date account of the prevalence of mental illness among nursing home residents. The review’s eligibility criteria include all mental disorders and clinically relevant symptoms, allowing for a broad consideration of mental illness in the target population. Restrictions are placed on study design and the measurement tools applied to ensure the highest quality evidence is identified. Meta-analyses will be undertaken to provide robust prevalence estimates across a range of presentations and assess potential sources of heterogeneity. Key insights will be highlighted, including any observed changes in the prevalence of mental illnesses in nursing home residents since the last comprehensive systematic review. Implications for researchers, care providers, and policymakers will be noted.

### Supplementary Information

**Additional file 1﻿.** PRISMA-P 2015 Checklist.Checklist for the reporting of systematic review and meta-analysis protocols.

**Additional file 2.** Database Search Queries for a Systematic Review into the Prevalence of Mental Illness Among Nursing Homes Residents. Search queries for databases for a systematic review and metal-analysis of the prevalence of mental illness among nursing homes residents.

**Additional file 3.** Data Extraction Template. Data extraction template for a systematic review and metal-analysis of the prevalence of mental illness among nursing homes residents, adapted from the JBI Data Extraction Form for Prevalence Studies.

## Data Availability

Not applicable.

## Abbreviations

APA: American Psychological Association
CINAHL: Cumulated Index to Nursing and Allied Health Literature
DSM-5-TR: Diagnostic and Statistical Manual of Mental Disorders, Fifth Edition, Text Revision
GRADE: Grading of Recommendations, Assessment, Development, and Evaluations
JBI: Joanna Briggs Institute
MeSH: Medical Subject Headings
PRISMA: Preferred Reporting Items for Systematic Reviews and Meta-Analyses
PRISMA-P: Preferred Reporting Items for Systematic review and Meta-Analysis Protocols
PROSPERO: The International Prospective Register of Systematic Reviews
