# Supplementary material for: Protocol for a systematic review and meta-analysis of the prevalence of mental illness among nursing home residents
Source: Syst Rev. 2024 Apr 16;13:109. doi: 10.1186/s13643-024-02516-1 (PMC11020180; doi:10.1186/s13643-024-02516-1)
Supplement: Supplementary file 2 — Additional file 2. Database Search Queries for a Systematic Review into the Prevalence of Mental Illness Among Nursing Homes Residents. Search queries for databases for a systematic review and metal-analysis of the prevalence of mental illness among nursing homes residents. [file 13643_2024_2516_MOESM2_ESM.docx]

# Database Search Queries for a Systematic Review into the Prevalence of Mental Illness Among Nursing Homes Residents

**Table 1.** Keywords and synonyms to inform database search queries for the systematic review

| **Keywords** | **Synonym** |
| --- | --- |
| Mental illness | Mental disorders  Psychiatric disorders/illness  DSM-5-TR diagnoses (various) |
| Nursing homes | Homes for the aged  Long term care  Residential aged care  Aged care homes  Skilled nursing facility  Institutionalised elderly/older adults |
| Prevalence | Incidence  Epidemiology |

**Table 2.** Iteratively derived database search queries for selected databases

| **PubMed** | 1. ATM: ADHD OR Adjustment disorder OR Agoraphobia OR Akathisia OR Alcohol use disorder OR Amphetamine-related disorder OR Anorexia …   *NOTE: See Appendix A for the full list of terms used*   1. ATM: prevalence OR incidence OR epidemiology 2. ATM: homes for the aged OR nursing homes OR "long-term care" 3. #1 AND #2 AND #3 AND (2009:2023[pdat])   *NOTE: The search used PubMed’s automatic term mapping (ATM) function to ensure a comprehensive search. ATM operates to automatically include relevant MeSH terms and equivalent free-text search terms. For example, the ATM of ‘prevalence’ within query #2 includes: "epidemiology"[MeSH Subheading] OR "epidemiology"[All Fields] OR "prevalence"[All Fields] OR "prevalence"[MeSH Terms] OR "prevalance"[All Fields] OR "prevalences"[All Fields] OR "prevalence s"[All Fields] OR "prevalent"[All Fields] OR "prevalently"[All Fields] OR "prevalents"[All Fields]* |
| --- | --- |
| **EMBASE (Ovid)**  *NOTE: terms followed by ‘/’ are MeSH terms* | 1. exp mental disease/ 2. (ADHD or "Adjustment disorder*" or Agoraphobi* or Akathisi* or "Alcohol use disorder*" or "Amphetamine-related disorder*" or Anorexi* … ).mp.   *NOTE: See Appendix A for the full list of terms used*   1. exp prevalence/ 2. exp incidence/ 3. exp nursing home/ 4. exp home for the aged/ 5. exp institutionalized elderly/ 6. exp residential care/ 7. long term care.mp. 8. 1 or 2 9. 3 or 4 10. 5 or 6 or 7 or 8 or 9 11. 10 and 11 and 12 12. limit 13 to yr="2009 -Current" |
| **Web of Science (Clarivate)** | 1. TS=(ADHD or "Adjustment disorder*" or Agoraphobi* or Akathisi* or "Alcohol use disorder*" or "Amphetamine-related disorder*" or Anorexi* … … or Trichotillomani* or "Use disorder*" or "Voyeuristic disorder*")   *NOTE: See Appendix A for the full list of free-text terms*   1. ALL=(prevalenc* OR incidence OR epidemiolog*) 2. TS=("long term care” OR "aged care” OR "skilled nursing facilit*” OR "nursing home*” OR "homes for the aged” OR "institutionali?ed elderly” OR "institutionali?ed older people") 3. #1 AND #2 AND #3 AND PY=(2009-2099) |
| **PsycInfo**  **(APA PsycNet)** | 1. Index Terms: {Mental Disorders} OR {Affective Disorders} OR {Anxiety Disorders} OR {Behavior Disorders} OR {Bipolar Disorder} OR {Borderline States} OR {Chronic Mental Illness} OR {Dissociative Disorders} OR {Eating Disorders} OR {Gender Dysphoria} OR {Mental Disorders Due to General Medical Conditions} OR {Neurocognitive Disorders} OR {Neurodevelopmental Disorders} OR Neurosis OR {Obsessive Compulsive Disorder} OR Paraphilias OR {Personality Disorders} OR Psychosis OR {Serious Mental Illness} OR {Sleep Wake Disorders} OR {Somatoform Disorders} OR {Stress and Trauma Related Disorders} OR {Substance Related and Addictive Disorders} OR {Thought Disorders} OR {Treatment Resistant Disorders} OR {Transdiagnostic Treatment} OR {Toxic Disorders} OR {Abnormal Psychology} OR {Attention Deficit Disorder} OR {Attention Deficit Disorder With Hyperactivity} OR {Consciousness Disturbances} OR {Diagnostic and Statistical Manual} OR {Emotional Disturbances} OR Etiology OR {International Classification of Diseases} OR {Intellectual Development Disorder} OR {Learning Disorders} OR {Memory Disorders} OR {Mental Health and Illness Assessment} OR {Mental Health Screening} OR {Mental Illness (Attitudes Toward)} OR {Mental Status} OR {Narcissism} OR {Perceptual Disturbances} OR {Psychiatric Patients} OR {Psychiatric Symptoms} OR {Psychodiagnosis} OR {Psychological Assessment} OR Psychopathology OR Suicide OR {Structured Clinical Interview} OR   Any Field: ADHD OR "Adjustment disorder*" OR Agoraphobi* OR Akathisi* OR "Alcohol use disorder*" OR "Amphetamine-related disorder*" OR Anorexi* …  *NOTE: See Appendix A for the full list of terms used*   1. Any Field: "prevalen*" OR "incidence" OR "epidemiolog*" 2. Index Terms: {Long Term Care} OR {Nursing Home Residents} OR {Nursing Homes} OR {Residential Care Institutions} OR   Any Field: “long term care” OR “residential aged care” OR “aged care home*” OR “skilled nursing facilit*” OR “nursing home*” OR “homes for the aged” OR “institutionali?ed elderly” OR “institutionali?ed older people”   1. Year: 2009 To 2099 2. #1 AND #2 AND #3 AND #4   *NOTE: Step 5 was executed by navigating to the ‘Recent Searches’ page and using the ‘Combine Searches’ function. The syntax provided for Step 5 is representative, but not operational.* |
| **CINAHL (EBSCOhost)** | 1. (MH "Behavioral and Mental Disorders+") 2. ADHD or "Adjustment disorder*" or Agoraphobi* or Akathisi* or "Alcohol use disorder*" or "Amphetamine-related disorder*" or Anorexi* …   *NOTE: See Appendix A for the full list of terms used*   1. TX prevalen* OR TX incidence OR TX epidemiolog* 2. "long term care" OR "aged care" OR "skilled nursing facilit*" OR "nursing home*" OR "homes for the aged" OR "institutionali?ed elderly" OR "institutionali?ed older people" 3. S1 OR S2 4. S3 AND S4 AND S5 5. S3 AND S4 AND S5 [Published Date: 20090101-20231231]   *NOTE: S7 was executed by navigating to the ‘View Results’ page for S6 and using the ‘Refine Results’ function to limit the publication date from 2009 to 2023, inclusive. The syntax provided for S7 is representative, but not operational.* |
| **Abstracts in Social Gerontology (EBSCOhost)** | 1. ADHD OR "Adjustment disorder*" OR Agoraphobi* OR Akathisi* OR "Alcohol use disorder*" OR "Amphetamine-related disorder*" OR Anorexi* …   *NOTE: See Appendix A for the full list of terms used*   1. TX prevalenc* or TX incidence or TX epidemiolog* 2. "long term care" OR "aged care" OR "skilled nursing facilit*" OR "nursing home*" OR "homes for the aged" OR "institutionali?ed elderly" OR "institutionali?ed older people" 3. S1 AND S2 AND S3 4. S1 AND S2 AND S3 [Published Date: 20090101-20231231]   *NOTE: S5 was executed by navigating to the ‘View Results’ page for S4 and using the ‘Refine Results’ function to limit the publication date from 2009 to 2023, inclusive. The syntax provided for S5 is representative, but not operational.* |

# Appendix A – Full Search Terms for Mental Disorders

The following search terms for mental disorders were produced by inspection of all mental disorder categories contained in the DSM-5-TR.

## Terms used for all databases except PubMed

ADHD OR "Adjustment disorder*" OR Agoraphobi* OR Akathisi* OR "Alcohol use disorder*" OR "Amphetamine-related disorder*" OR Anorexi* OR "Antisocial personality disorder*" OR Anxiety OR ASD OR "Attention-deficit/hyperactivity disorder*" OR "Autism" OR "Avoidant personality disorder*" OR "Binge-eating disorder*" OR Bipolar OR "Body dysmorphic disorder*" OR "Borderline personality disorder*" OR BPD OR Bulimi* OR "Catatonic disorder*" OR "Communication disorder*" OR "Conduct disorder*" OR "Conversion disorder*" OR "Cyclothymic disorder*" OR "Delusional disorder*" OR "Dependent personality disorder*" OR "Depersonalization/derealization disorder*" OR Depression OR Depressive OR "Developmental coordination disorder*" OR "Disinhibited social engagement disorder*" OR "Disruptive mood dysregulation disorder*" OR "Dissociative disorder*" OR "Dissociative identity disorder*" OR Dyskinesi* OR Dysthymi* OR Dystoni* OR "Eating disorder*" OR "Elimination disorder*" OR Encopresis OR Enuresis OR "Excoriation disorder*" OR "Exhibitionistic disorder*" OR "Factitious disorder*" OR "Feeding disorder*" OR "Female orgasmic disorder*" OR "Female sexual interest/arousal disorder*" OR "Fetishistic disorder*" OR "Fluency disorder*" OR "Food intake disorder*" OR "Frotteuristic disorder*" OR GAD OR "Gambling disorder*" OR "Gender dysphori*" OR "Generalized anxiety disorder*" OR "Genito-pelvic pain/penetration disorder*" OR "Hair-pulling disorder*" OR "Hallucinogen persisting perception disorder*" OR "Histrionic personality disorder*" OR "Hoarding disorder*" OR "Hypersomnolence disorder*" OR "Illness anxiety disorder*" OR "Insomnia disorder*" OR "Intellectual developmental disorder*" OR "Intermittent explosive disorder*" OR Kleptomani* OR "Language disorder*" OR "Male hypoactive sexual desire disorder*" OR MDD OR "Mental disorder*" OR "Mental illness*" OR "Movement disorder*" OR "Narcissistic personality disorder*" OR "Neurocognitive disorder*" OR "Neurodevelopmental disorder*" OR "Nightmare disorder*" OR NPD OR Obsessive-compulsive OR OCD OR "Oppositional defiant disorder*" OR "Panic disorder*" OR "Paranoid personality disorder*" OR "Paraphilic disorder*" OR "Pedophilic disorder*" OR "Personality disorder*" OR Phobi* OR Pica OR "Premenstrual dysphoric disorder*" OR "Psychiatric disorder*" OR "Psychiatric illness*" OR "Psychotic disorder*" OR PTSD OR Pyromani* OR "Reactive attachment disorder*" OR "Restless legs syndrome" OR "Rumination disorder*" OR Schizo* OR "Selective mutism" OR "Self-harm" OR "Separation anxiety disorder*" OR "Sexual dysfunction" OR "Sexual masochism disorder*" OR "Sexual sadism disorder*" OR "Skin-picking disorder*" OR "Sleep arousal disorder*" OR "Sleep behaviour disorder*" OR "Sleep disorder*" OR "Sleep-wake disorder*" OR "Social anxiety disorder*" OR "Somatic symptom disorder*" OR "Specific learning disorder*" OR "Speech sound disorder*" OR "Stereotypic movement disorder*" OR "Stimulant-related disorder*" OR "Stress disorder*" OR "Stress-related disorder*" OR "Tic disorder*" OR "Tobacco-related disorder*" OR "Tourette*" OR "Transvestic disorder*" OR "Trauma-related disorder*" OR Trichotillomani* OR "Use disorder*" OR "Voyeuristic disorder*"

NOT INCLUDED: Unspecified alcohol-related disorder; Unspecified caffeine-related disorder; Unspecified cannabis-related disorder; Unspecified cocaine-related disorder; Unspecified hallucinogen-related disorder; Unspecified inhalant-related disorder; Unspecified opioid-related disorder; Unspecified phencyclidine-related disorder; Unspecified stimulant-related disorder

## Terms used for PubMed

PubMed uses predefined phrases to translate search terms across its databases. However, several terms in the list on the previous page were found to be undefined, irrelevantly defined, or redundant. For example, frotteuristic disorder was undefined; PTSD was irrelevantly defined as ‘6‑pyruvoyl-tetrahydropterin synthase deficiency’; and mental disorder, mental illness, psychiatric disorder, and psychiatric illness all translate to the same search terms. As a result, the number of search terms used to search PubMed was slightly shorter than those used for other databases.

ADHD OR Adjustment disorder OR Agoraphobia OR Akathisia OR Alcohol use disorder OR Amphetamine-related disorder OR Anorexia OR Antisocial personality disorder OR Anxiety OR Attention-deficit/hyperactivity disorder OR Autism OR Avoidant personality disorder OR Binge-eating disorder OR Bipolar disorder OR Body dysmorphic disorder OR Borderline personality disorder OR Bulimia OR Catatonic OR Communication disorder OR Conduct disorder OR Conversion disorder OR Cyclothymic disorder OR Delusional disorder OR Dependent personality disorder OR Depersonalization/derealization OR Depression OR Developmental coordination disorder OR “Disinhibited social engagement disorder” OR “Disruptive mood dysregulation disorder” OR Dissociative disorder OR Dissociative identity disorder OR Dyskinesia OR Dysthymia OR Dystonia OR Eating disorder OR Elimination disorder OR Encopresis OR Enuresis OR “Excoriation disorder” OR “Exhibitionistic disorder” OR Factitious disorder OR Feeding disorder OR “Female orgasmic disorder” OR “Female sexual interest/arousal disorder” OR Fetishistic disorder OR “Fluency disorder” OR “Food intake disorder” OR Gambling disorder OR Gender dysphoria OR “Generalized anxiety disorder” OR “Generalised anxiety disorder” OR “Genito-pelvic pain/penetration disorder” OR “Hallucinogen persisting perception disorder” OR Histrionic personality disorder OR Hoarding disorder OR Hypersomnolence disorder OR “Illness anxiety disorder” OR Insomnia disorder OR Intellectual developmental disorder OR Intermittent explosive disorder OR Kleptomania OR Language disorder OR hypoactive sexual desire disorder OR Mental disorder OR “Mental illness” OR Movement disorder OR Narcissistic personality disorder OR Neurocognitive disorder OR Neurodevelopmental disorder OR “Nightmare disorder” OR Obsessive-compulsive disorder OR OCD OR Oppositional defiant disorder OR Panic disorder OR Paranoid personality disorder OR Paraphilic disorder OR Pedophilic OR Personality disorder OR Phobia OR Pica OR Posttraumatic stress disorder OR Premenstrual dysphoric disorder OR “Psychiatric disorder” OR “Psychiatric illness” OR Psychotic disorder OR Pyromania OR Reactive attachment disorder OR Restless legs syndrome OR Rumination disorder OR Schizophrenia OR Schizoaffective disorder OR schizoid personality disorder OR Schizophreniform disorder OR Schizotypal personality disorder OR Selective mutism OR Self-harm OR Separation anxiety disorder OR Sexual dysfunction OR Sexual masochism disorder OR Sexual sadism disorder OR “Skin-picking disorder” OR Sleep arousal disorder OR “Sleep behaviour disorder” OR “Sleep behavior disorder” OR Sleep disorder OR Sleep-wake disorder OR Social anxiety disorder OR “Somatic symptom disorder” OR Specific learning disorder OR Speech sound disorder OR Stereotypic movement disorder OR (Stress disorder OR “Stress-related disorder” OR Tic disorder OR Tourette’s OR “Trauma-related disorder” OR Trichotillomania
