# Supplementary material for: Protocol for a systematic review and meta-analysis of the prevalence of mental illness among nursing home residents
Source: Syst Rev. 2024 Apr 16;13:109. doi: 10.1186/s13643-024-02516-1 (PMC11020180; doi:10.1186/s13643-024-02516-1)
Supplement: Supplementary file 3 — Additional file 3. Data Extraction Template. Data extraction template for a systematic review and metal-analysis of the prevalence of mental illness among nursing homes residents, adapted from the JBI Data Extraction Form for Prevalence Studies. [file 13643_2024_2516_MOESM3_ESM.docx]

# Data Extraction Template for Systematic Review and Meta-Analysis^[[1]](#footnote-1)^

| **Citation Details** | Rayyan ID |  |
| --- | --- | --- |
|  | Data extraction date |  |
|  | Authors |  |
|  | Title |  |
|  | Year |  |
|  | Journal / publisher |  |
| **Study Methods** | Study design |  |
|  | Year/s of data collection |  |
|  | Location (country [region[) |  |
|  | Sample size (n) |  |
|  | Age (mean) |  |
|  | Age (std. dev.) |  |
|  | Age (other) |  |
|  | Gender distribution (n_fem_) |  |
|  | Primary outcome measure/s |  |
|  | Mental illness/es measured |  |
|  | Diagnostic criteria / instrument used |  |
| **Study Results** | Prevalence rate (n_MI_) |  |
|  | Prevalence rate by gender (nMI*f) |  |
|  | Prevalence rate by gender (nMI*m) |  |
|  | Risk of bias rating (based on JBI) |  |
|  | Reviewer comments (e.g. bias/ethical considerations) |  |

1. Adapted from the ‘Data Extraction Form for Prevalence Studies’ contained in Munn Z, Moola S, Lisy K, Riitano D, Tufanaru C. Chapter 5: Systematic reviews of prevalence and incidence. In: Aromataris E, Munn Z (Editors). *JBI Manual for Evidence Synthesis*. JBI, 2020. Available from <https://synthesismanual.jbi.global> <https://doi.org/10.46658/JBIMES-20-06>

   [↑](#footnote-ref-1)
